
JOURNAL INFORMATION
==============================
NLM Title Abbreviation: ScientificWorldJournal
Journal ID: ScientificWorldJournal
NLM Journal ID: 101131163
Journal ID: TSWJ

The Scientific World Journal

ISSN: 2356-6140
EISSN: 1537-744X
Publisher: Wiley

ARTICLE INFORMATION
==============================
PMCID: PMC6083919
PMID: 30147525
DOI: 10.1100/tsw.2001.175
Article ID: 280151
Article version: 1
Subjects: Short Report

Changes in Intracellular Adp:Atp Ratios as a Marker of Apoptosis

Simmons Tracy D. *
Slater Kevin J.
Crouch Sharon P.M.
LumiTech (UK) Ltd, Nottingham Business Park City Link, Nottingham, NG2 4LA, UK
*Tracy D. Simmons: t.simmons@lumitech.co.uk
Publication date: 2001
Electronic publication date: 2001 Aug 11
Volume: 1
First page: 58
Last page: 58
Copyright: Copyright © 2001 Tracy D. Simmons et al.
Copyright year: 2001
License: This is an open access article distributed under the Creative Commons Attribution License, which permits unrestricted use, distribution, and reproduction in any medium, provided the original work is properly cited.
License URL: https://creativecommons.org/licenses/by/3.0/

==============================
Miami *Nature Biotechnology* Short Reports
*TheScientificWorld* (2001) 1(S3), 58SR
ISSN 1532-2246; DOI 10.1100/tsw.2001.175

### CHANGES IN INTRACELLULAR ADP:ATP RATIOS AS A MARKER OF APOPTOSIS

Tracy D. Simmons*, Kevin J. Slater, and Sharon P.M. Crouch

LumiTech (UK) Ltd, Nottingham Business Park City Link, Nottingham, NG2 4LA, UK
* t.simmons@lumitech.co.uk

**INTRODUCTION.** Oxidative phosphorylation occurs across the inner mitochondrial membrane, resulting in the synthesis of ATP and maintenance of the mitochondrial transmembrane potential ($\Delta\Psi_m$). One of the earliest events in apoptosis is believed to be the breakdown in oxidative phosphorylation (1) and subsequently a decrease in ATP and loss of $\Delta\Psi_m$. We have shown in a number of apoptotic models, that the ADP:ATP ratio can be used reliably as a screen for both early and late apoptosis (2) in human leukaemic cell lines. We have now investigated the effect of the broad spectrum caspase inhibitor, Z-VAD-FMK, on determination of apoptosis by measurement of ADP:ATP ratios.

**METHOD.** HL-60 cells were incubated with increasing concentrations camptothecin (CAM). Time course experiments were also carried out in the presence/absence of 5μM CAM, and apoptosis was determined every 30 minutes for up to 8 hours. The bioluminescent detection of ADP:ATP ratios was determined using the ApoGlow kit (LumiTech (UK) Ltd). Apoptosis was confirmed by Romanowsky staining, and determination of the percentage of hypodiploid cells by propidium iodide (PI) staining and flow cytometry. Collapse of the $\Delta\Psi_m$ was measured using the lipophilic cationic fluorescent probe JC-1. Increasing concentrations of Z-VAD-FMK were added to the cells either prior to addition of the drug, or at increasing time points after CAM to determine at which point the cells could still be ‘rescued’ from apoptosis.

**RESULTS.** There was a concentration dependent increase in ADP:ATP ratio that correlated with an increase in the hypodiploid population and a decrease in % J-aggregates ($p<0.0001$). The time course experiments with 5μM CAM showed the drop in ATP corresponding to a loss of $\Delta\Psi_{m,}$ and the increased ratios occurring concomitantly with DNA fragmentation. Cell membrane integrity was maintained for approximately 4 hours after DNA fragmentation was first detected, together with a significant increase in ADP:ATP ratio. After approximately 6.5 hours there was a loss of membrane integrity that correlated with a dramatic increase in ratios. Preliminary washing experiments showed that even when the CAM had been washed from the cells immediately after addition, further incubation for 4 hours still resulted in apoptosis. Addition of 50μM Z-VAD-FMK prior to the addition of CAM showed prevention of apoptosis after 4 hours incubation (see figure). Full protection was no longer observed when the inhibitor was added after 30 minutes incubation with CAM or at concentrations below 40μM.

**DISCUSSION.** Bioluminescent determination of ADP:ATP ratios correlated well with a number of other techniques used for detection of apoptosis. The correlation with loss of the $\Delta\Psi_m$ suggested the change in ratios to be a good indicator of early apoptosis, in the model tested. The use of Z-VAD-FMK reduced ratios back to those of the control cells. As this compound is a broad spectrum inhibitor that appears to act on those caspases associated with early apoptotic events, this again confirms the change in ratios as a sensitive, quick and simple method for the determination of early apoptotic events.

**REFERENCES.**

1. Tsujimoto, Y. (1997) Cell Death Differ. 4, 429-434
2. Bradbury, D.A., Simmons, T.D., Slater, K.J., and Crouch, S.P.M. (2000) J. Immunol. Methods 240, 79-92
